# The roles of T cells in obese adipose tissue inflammation

**DOI:** 10.1080/21623945.2021.1965314

**Published:** 2021-09-13

**Authors:** Qiong Wang, Yurong Wang, Danyan Xu

**Affiliations:** a Department of Cardiovascular Medicine, The Second Xiangya Hospital, Central South University, Changsha, Hunan, China; b Department of Internal Cardiovascular Medicine, Second Xiangya Hospital, Central South University, Changsha, Hunan, China

**Keywords:** Adipose tissue inflammation, cd4^+^ t cells, cd8^+^ t cells, treg cells

## Abstract

Adipose tissue inflammation in obese patients can cause a series of metabolic diseases. There are a variety of immune cells in adipose tissue, and studies have shown that T cells are associated with adipose tissue inflammation. This review aims to describe the current understanding of the relationship between T cells and adipose tissue inflammation, with a focus on regulation by T cell subtypes. Studies have shown that Th1, Th17 and CD8^+^ T cells, which are important T cell subsets, can promote the development of adipose tissue inflammation, whereas Treg cells protect against inflammation, suggesting that targeting the mechanism by which T cell subtypes regulate adipose tissue inflammation is a potential therapeutic strategy for treating obesity. T cells play important roles in regulating obesity-associated adipose tissue inflammation, thus providing new research directions for the treatment of obesity. More studies are needed to clarify how T cell subtypes regulate adipose tissue inflammation to identify new treatments for obesity.

Obesity, considered a chronic low-grade inflammatory disease, is a systemic metabolic syndrome caused by adipocyte hypertrophy and an increase in adipose tissue (AT) [1]. In 1993, Hotamilligil et al. [2] first proposed the concept of AT inflammation. AT is a major metabolic organ that stores excess fat and an important endocrine organ that regulates the balance of energy intake and consumption by secreting soluble factors, including adipokines, chemokines and cytokines [3]. AT inflammation is caused by the excessive production of inflammatory cytokines and chemokines and products of adipocyte death to promote inflammatory cell accumulation and activation in AT in obese patients [4].

AT inflammation has emerged as a major process linking obesity and its associated pathology. AT plays a crucial role as the source and site of inflammation. Studies have shown that the AT of obese patients is also a site of significant immune cells accumulation [5]. Initially, Weisberg et al. [6] showed that macrophages were the main culprit in most AT inflammatory events. Subsequent studies have shown that T cells also accumulate in AT and are involved in AT inflammation when the phenotype of obesity is activated [7]. Recent studies induced T cell activation by feeding mice a high-fat diet (HFD), which increased proinflammatory cytokine production by CD4^+^ and CD8^+^ T cells [8], further demonstrating the important role of T cells in obesity. Based on the correlation between these two phenomena, this review briefly analyzes and summarizes the roles and mechanisms of T cells in AT inflammation and explores new ideas for the treatment of obesity.

## 1. T cells and adipose tissue inflammation in obesity

The accumulation of immune cells and the expression of proinflammatory cytokines and chemokines are characteristics of AT inflammation [9]. Compared with subcutaneous AT, visceral adipose tissue (VAT) contains more immune cells and plays a more critical role in immune metabolic homoeostasis [10]. The main immune cell types in VAT include macrophages and T cells [7]. Recent studies have shown that the changes in T cell components residing in inflamed AT are associated with the degree of obesity-induced AT inflammation [11]. T cells are divided into cytotoxic CD8^+^ T cells that recognize major histocompatibility complex (MHC) I-presented antigens and CD4^+^ T cells that interact with MHC II-presented antigens [12]. CD4^+^ T cells can be further divided into regulatory T (Treg) cells and T helper (Th) cells, and Th cells can be separated into three main subsets: Th1, Th2 and Th17 cells [13]. Studies have shown increased T cell infiltration of VAT in both obese humans and obese mice [14]. In the process of obesity-induced VAT inflammation, the relative balance between proinflammatory T cells and anti-inflammatory T cells is changed. The pool of proinflammatory T cells, such as CD4^+^ T and CD8^+^ T cells, is increased, as is their secretion of proinflammatory cytokines, thus promoting the development of AT inflammation. At the same time, the decrease in anti-inflammatory T cells such as Treg cells and the corresponding decrease in the inhibitory effect of these cells on inflammation aggravate inflammation [15]. The T cell subsets in AT are closely associated with the development of AT inflammation. (Figure)

**Figure:**
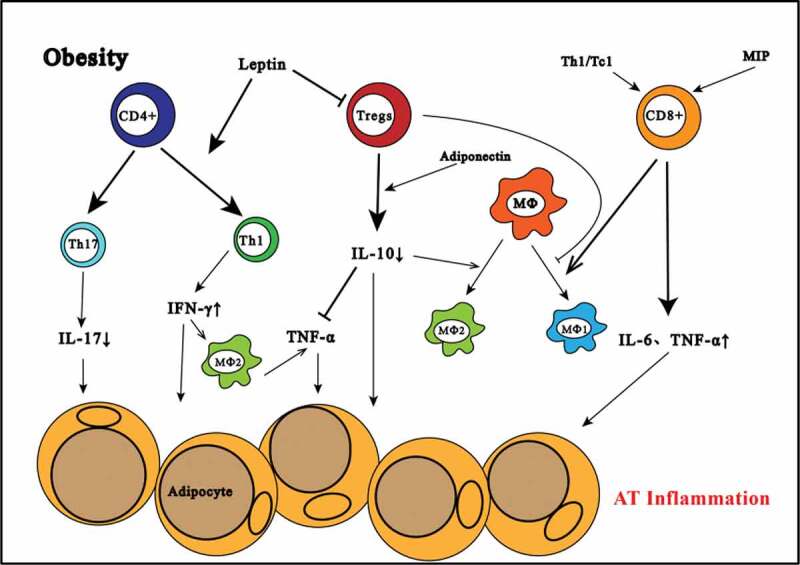
Immune cells regulate inflammation in obesity adipose tissue; Treg, regulatory T; IFN-γ, interferon-γ; MCP-1, monocyte chemoattractant protein-1; Th,T helper;Tc,T cytotoxic;TNF-α,tumor necrosis factor-α;MΦ, macrophage; AT,adipose tissue

## 2. CD4^+^ T cells

As one of the CD4^+^ T cell subsets, Th cells are an important component of adaptive immunity and participate in a variety of immune processes, from macrophage activation to cytotoxic T cell activation. Th1 cells mainly secrete interferon-gamma (IFN-γ), Th2 cells produce the anti-inflammatory cytokine IL-10 [16], and the main cytokine produced by Th17 cells is IL-17 [17].

### 2.1 The role of Th1 cells in AT inflammation

Pacifico et al. [18] found an increased number of Th1 cells and greater IFN-γ secretion in the context of obesity. As early as 1992, studies showed that IFN-γ, the iconic cytokine of Th1 cells, could coordinate the inflammatory cascade [19]. Later studies showed that IFN-γ could induce the production of inflammatory mediators in macrophages; induce the expression of leukocyte adhesion molecules, chemokines and MHC II molecules; and increase the antigen-presenting abilities of macrophages and endothelial cells [19], thereby promoting the occurrence of inflammation. This finding suggests that Th1 cells play an important role in regulating inflammation in AT. Similarly, Zhang et al. [20] showed that IFN-γ not only induced AT inflammation but also increased macrophage accumulation, and the excessive accumulation of macrophages in obese AT further promoted AT inflammation. Therefore, the increase in macrophages induced by greater IFN-γ production further exacerbates the inflammation. More importantly, further studies have shown that AT-derived IFN-γ is a key cytokine that drives the transformation of macrophages from anti-inflammatory M2 to proinflammatory M1 cells [21]. Proinflammatory macrophages secrete inflammatory cytokines that exacerbate AT inflammation, trigger the production of additional inflammatory cytokines such as tumour necrosis factor-α (TNF-α), and stimulate adipocytes to produce chemokines, including IFN-γ-inducible protein (IP)-10, monocyte chemotactic protein-1 (MCP)-1 and IL-6 [22]. In contrast, Winer et al. [23] showed that reductions in Th1 cells and IFN-γ secretion in AT led to decreased AT inflammation and increased insulin sensitivity. Furthermore, IFN-γ-knockout obese mice showed decreased fat cell volume and decreased inflammatory cell accumulation in AT, which was accompanied by an improvement in insulin sensitivity [24]. This finding clearly confirms the important role of Th1 cells in AT inflammation.

Therefore, understanding the mechanism of Th1 cells in AT inflammation will provide directions for the treatment of obesity. In diet-induced obese (DIO) mice, CD4^+^ T cells in VAT were activated by adipocytes and adipocyte-derived leptin, and OX40 protein was expressed on activated CD4^+^ T cells, which has been shown to promote T cell differentiation, expansion and cytokine production [25]. Liu et al. [26] showed that the expression of OX40 and adipocyte-derived leptin jointly promoted the proliferation and differentiation of CD4^+^ T cells to Th1 cells in HFD mice, which significantly increased the number of Th1 cells and induced immune-associated inflammation. This increased Th1 cell population produced a large amount of IFN-γ, which promotes M1 polarization of AT macrophages; these M1 macrophages subsequently secreted proinflammatory cytokines that promote AT inflammation and insulin resistance. However, when the OX40 gene was deleted, the numbers of Th1 cells and M1 macrophages were significantly reduced, resulting in decreased adipocyte volume and AT weight. In addition, Ren et al. [27] showed that the transformation-associated protein 73 (p73) gene, which is a member of the p53 family, is a potential regulator of Th1 cell differentiation that inhibits Th1 cell differentiation and limits the production of inflammatory factors by inhibiting target genes in Th1 cells. Moreover, p73 can directly bind to the IFN-γ gene to inhibit IFN-γ production, thus blocking the development of inflammation. Therefore, targeted inhibition of OX40 protein and the p73 gene, which are involved in regulating the proinflammatory effects of Th1 cells, provides a possibility for the treatment of obesity.

### 2.2 The role of Th17 cells in adipose tissue inflammation

IL-17, the signature cytokine of Th17 cells, is a powerful proinflammatory mediator. Numerous studies have shown that Th17 cells are associated with obesity-induced inflammation [28]. Schindler et al. [29] showed that the proportion of circulating Th17 cells was significantly higher in overweight children than in the normal control group, and the proportion of Th17 cells was positively correlated with body mass index (BMI). In addition, they found that the mRNA expression of orphan receptor C (RORC) and IL-17A was significantly increased; RORC is an important transcription factor in Th17 cells. These findings suggest that Th17 cells and IL-17 may be involved in AT inflammation. Furthermore, studies showed that IL-17 receptor knockout significantly reduced the body and visceral fat pad weights of mice fed a HFD and improved AT inflammation and insulin sensitivity [30]. Similarly, Lu et al. [31] neutralized the inflammatory factor IL-17 with IL-17 antibodies and subsequently found reduced epididymal AT inflammation and macrophage accumulation. This finding demonstrates the important role of IL-17 in promoting obesity-associated AT inflammation. In addition, studies using IL-17-deficient mice indicated that IL-17 may cause systemic inflammation long before the onset of obesity, thus making individuals susceptible to metabolic diseases [32]. More importantly, Gaffen et al. [33] showed that IL-17 may contribute to the recruitment of immune cells because IL-17 can induce the expression of various chemokines (such as MCP-1, MCP-3, IP-10, and MIG) that are essential for leukocyte tissue infiltration, which may further exacerbate AT inflammation. In addition, studies have shown that inhibition of TANK-binding kinase 1 (TBK1), a key regulator of the inflammatory response, can reduce IL-17-induced inflammation in obese mice, suggesting that IL-17 regulates AT inflammation in a TBK1-dependent manner [34].

However, there are puzzling data showing that IL-17, in addition to its proinflammatory properties, can delay the development of obesity by inhibiting precursor transcription factors and adipokines and acting on preadipocytes to inhibit adipogenesis and differentiation [32]. Zúñiga et al. [32] exposed 3T3-L1 preadipocytes to adipogenic conditions with or without IL-17 for 2 days and found that the presence of IL-17 significantly inhibited lipid uptake and the expression of mature adipocyte-related genes during differentiation. In the absence of IL-17, 3T3-L1 cells differentiated well, resulting in upregulated mRNA expression of adipogenic transcription factors, adipocyte-related cytokines and lipid-related genes. In addition, studies have shown that Th17 cells can adhere to mesenchymal stem cells under inflammatory conditions and adopt Treg-like phenotypes [35]; Treg cells have anti-inflammatory properties, thus contradicting the proinflammatory properties of IL-17 secreted by Th17 cells. Similarly, recent studies have shown that IL-25, a member of the IL-17 family, can promote M2 macrophage polarization and regulate enzymes associated with promoting lipolysis and inhibiting lipogenesis in macrophages and adipocytes, thereby promoting lipid metabolism and energy production, alleviating lipid accumulation in the liver and AT and reducing the increase of obesity [36]. These findings further confirm the anti-inflammatory effects of IL-17.

Researchers have proposed different explanations for the above mentioned differences in the effects of IL-17 in various studies. In the study by Hong et al. [37], purified Treg cells and Th17 cells were coinjected into HFD-fed mice to prevent Th17-mediated inflammation. The mice injected with Th17 and Treg cells showed reductions in body weight and body fat, a significant reversal in HFD-induced liver lipid accumulation, and improved glucose tolerance and insulin sensitivity. However, the control group that received Treg cells alone or phosphate-buffered saline (PBS) did not show any effects. Moreover, the researchers showed that IL-17 is the key molecule underlying the abovementioned role of Th17 cells. This finding further confirms the important role of IL-17 in delaying obesity. The researchers posited that these results contradict the observed proinflammatory role of IL-17 in obesity because the above mentioned studies focused on Th17 cells in the mouse intestine, which are unique and different from other populations of Th17 cells, such as those in AT, which are increased by inflammatory stimulation. In addition, Jung et al. [38] showed that IL-17 plasma levels were decreased significantly in overweight adolescents without atherosclerosis, insulin resistance or other metabolic syndromes. Previous studies have shown that IL-17 can delay the progression of obesity, suggesting that the findings of Jung et al. may be due to decreased IL-17 leading to a weakened inhibitory effect on adipogenesis and adipocyte differentiation, which leads to obesity in adolescents; this proposal supports the above mentioned effects of IL-17. As an explanation of the contradictory results regarding the proinflammatory properties of IL-17, the author notes that in his study, IL-17 levels were detected in circulation but not in subcutaneous AT or VAT. Therefore, the author believes that his findings do not contradict the elevated IL-17 level in the AT of obese patients. Although these studies have attempted to explain the different roles of IL-17 in obese individuals, no studies have systematically clarified these discrepancies. Therefore, further studies are needed to explain these differences to provide more possibilities for the treatment of obesity.

### 2.3 The role of regulatory T cells in adipose tissue inflammation

As one of the subsets of CD4^+^ T cells, Treg cells participate in the regulation of AT inflammation. Treg cells can originate in the thymus or outside the thymus. Initial studies suggested that Treg cells account for only a small portion of peripheral T cells and make up approximately 5–15% of CD4^+^ T cell subsets. Later studies showed large numbers of CD4^+^FoxP3^+^ Treg cells in VAT, and these cells accumulated with age in lean mice [39]. In mice that underwent long-term high-caloric feeding or in mice with hereditary obesity, the number of Treg cells in VAT gradually decreased with age [39]. In obese patients, the number of Treg cells was significantly reduced, while the proportion of proinflammatory monocytes was increased [40], suggesting that Treg cells play a role in obese patients. Further studies have shown that the decrease in Treg cells in AT leads to a stronger proinflammatory environment and promotes the development of type 2 diabetes [41]. Similarly, Eller et al. [42] showed that the significant decrease in the number of Treg cells in VAT was closely associated with the increase in inflammatory mediators and the decrease in insulin sensitivity in VAT. To demonstrate the indispensable effect of Treg cells in AT, these cells were depleted through multiple methods, and the results showed that the loss of Treg cells led to worsened metabolic parameters, such as elevated fasting glucose levels and impaired insulin sensitivity. In contrast, supplementation with Treg cells reduced VAT inflammation and improved metabolic parameters in obese mice [43]. In addition, studies [44] have shown that Treg cells in VAT exhibit sex differences. Comparisons of Treg cells in the VAT of males and females revealed significant differences in transcriptional profiles that were not seen in Treg cells in the spleen. Sex differences may account for different mechanisms of regulating inflammation in VAT. AT is rich in sex hormones. Interestingly, research has shown decreased VAT quality and improved glucose tolerance after androgen receptor knockout in mice, whereas oestrogen receptor knockout in mice increased VAT quality and decreased glucose tolerance. These results suggest that oestrogen reduces AT inflammation, while androgen promotes inflammation. However, further studies have identified specific stromal cells in male VAT that can produce IL-33 and mediate the expansion of Treg cells in a B lymphocyte-induced mature protein-1 (Blimp1)-dependent manner, thus limiting inflammation in VAT. These studies show that Treg cells play a protective role in obesity-induced AT inflammation, and exploring the mechanism and factors that regulate AT inflammation through Treg cells is expected to provide new strategies for the treatment of obesity.

#### 2.3.1 IL-10 plays a protective role in obesity-induced adipose tissue inflammation

IL-10 can protect against obesity-associated AT inflammation. Studies have shown that under normal conditions, Treg cells in VAT produce high levels of IL-10 and upregulate genes downstream of IL-10 receptors, thus producing an anti-inflammatory environment. In the context of obesity, the Treg cell population in AT decreases, resulting in decreased secretion of the anti-inflammatory cytokine IL-10 and leading to AT inflammation [39]. IL-10 levels may be negatively correlated with AT inflammation. Han et al. [45] showed that IL-10 production by Treg cells is necessary to inhibit production of the proinflammatory factor TNF-α by macrophages. Macrophages can express IL-10 receptors, and IL-10 plays an anti-inflammatory role by interacting with IL-10 receptors to activate anti-inflammatory signalling pathways. Therefore, Toita et al. [46] introduced IL-10 into macrophages by using phosphatidylserine liposomes (PSLs) as biological carriers; PSLs can transform inflammatory M1 macrophages into anti-inflammatory M2 macrophages, and IL-10 and PSLs had synergistic anti-inflammatory effects. IL-10, PSLs and IL-10-coupled PSLs (PSLs-IL10) were injected intraperitoneally into HFD-fed mice; in the IL-10 and PSLs-IL10 groups, the visceral fat weight of mice and the secretion of proinflammatory cytokines (IL-6 and TNF-α) decreased significantly, suggesting that IL-10 plays an important anti-inflammatory role in obese AT by acting on macrophages. In addition, studies have shown that IL-10 produced by Treg cells in VAT not only inhibits the release of TNF-α-induced inflammatory cytokines and chemokines but also protects adipocytes from the effects of TNF-α-induced insulin resistance [39]. Therefore, reduced IL-10 production stemming from a smaller Treg cell population may exacerbate obesity-associated AT inflammation, suggesting that IL-10 plays an important role in resisting AT inflammation. Wingless associated integration site 5A (Wnt5a) is a member of the atypical Wnt family. Previous studies have shown that inhibiting the expression of Wnt5a in 3T3-L1 cells prevents lipid accumulation in the cytoplasm and reduces the expression of adipogenic genes [47]. A recent study by Kim et al. [48] showed that IL-10 inhibited AT inflammation and lipid accumulation by inhibiting Wnt5a expression in 3T3-L1 preadipocytes. In contrast, the study by Medeiros et al. [49] on childhood obesity showed that IL-10 deficiency can lead to chronic inflammation in obesity. This finding further confirms that IL-10 plays an important anti-inflammatory role in AT.

The main role of insulin is to promote glucose uptake in adipocytes, hepatocytes and cardiomyocytes, and this role is mainly facilitated by the insulin receptor-mediated Akt pathway [50]. Notably, the Akt signalling pathway regulates Treg cell function and therefore also plays an important role in regulating inflammation. Akt activation specifically inhibits IL-10 production and reverses the ability of Treg cells to inhibit TNF-α production by macrophages. The Akt pathway is one mechanism through which insulin plays a role, but it is unclear whether insulin regulates Treg cells through this pathway. To investigate this question, Han et al. [45] cultured Treg cells with insulin in the presence or absence of the Akt inhibitor Akti1/2, the mTOR inhibitor rapamycin and the Akt pathway-independent kinase MEK1/2 and found that IL-10 was significantly increased in the Akt pathway inhibitor groups (Akti1/2 and rapamycin). This finding suggests that the inhibitory effect of insulin on IL-10 production depends on the Akt and mTOR pathways, and thereby, insulin exacerbates obesity-related AT inflammation. Obesity may lead to an overproduction of insulin. The excessive accumulation of insulin inhibits the proliferation of Treg cells, thus reducing IL-10 production, and the decreased number of Treg cells in obesity leads to decreased IL-10 secretion; thus, insulin further exacerbates the development of obesity-associated AT inflammation. In contrast, Toda et al. [51] reduced Akt expression by 70–80% through specific Akt1/Akt2 double-knockout in mice with mTOR defects. Costimulation with insulin and lipopolysaccharide (LPS) significantly inhibited the production of IL-10. Insulin was confirmed to induce the expression of IL-10 in AT macrophages and bone marrow-derived macrophages through the PI3K-Akt-mTOR pathway, thereby inhibiting hepatic gluconeogenesis. The differences in these studies may be due to the different cells targeted by insulin, leading to differential expression of IL-10, but they also illustrate that the pathway connecting insulin to IL-10 in AT is complicated.

Numerous studies have shown that IL-10 can play an anti-inflammatory role to reduce the incidence of obesity, but recent studies have demonstrated the opposite role of IL-10 in AT. After feeding 10-week-old mice a HFD for 6 weeks, Rajbhandari et al. [52] showed that compared with WT mice, IL-10^−/-^ mice had increased energy consumption, significantly decreased body weight, significant glucose tolerance and improved insulin resistance. IL-10 can be produced by a variety of immune cells. Beppu et al. [53] found that CD4^+^Foxp3^+^ Treg cells are an important source of IL-10 in this context and that Treg cell-derived IL-10 can inhibit adipocyte proliferation, adipocyte differentiation and AT thermogenesis. However, when Treg-specific IL-10 was knocked down, the expression of uncoupled protein 1 (UCP1) and other thermogenic genes in adipocytes in white AT was increased, protecting mice from insulin resistance and diet-induced obesity. In addition, this research group demonstrated that the secretion of IL-10 by Treg cells acts via Blimp-1, a transcription factor that is highly expressed in CD4^+^ T cells and directly regulates the expression of genes associated with T cell effectors and Treg function [54]. IL-10 is secreted by many immune cells and plays different roles. Although an increasing number of studies have shown that the abovementioned effects on AT are different from the anti-inflammatory effects, the reasons for this contradiction have not been clarified, and further research and elucidation of these contradictions are expected to provide targets for the treatment of obesity.

## 3. CD8^+^ T cells initiate and maintain adipose tissue inflammation

CD8^+^ T cells, also known as cytotoxic T cells, are activated by MHC-I antigens on the surface of antigen-presenting cells [55] and are involved in the recognition and clearance of damaged or dysfunctional cells. CD8^+^ T cells play an important role in the initiation and maintenance of AT inflammation and systemic insulin resistance. Previous studies have reported that the number of CD8^+^ T cells increases with increasing obesity and is usually three to four times higher in obese people than in lean people. Recent studies [56] have also indicated that mice fed a HFD have increased AT CD8^+^ T cell density. To directly examine the role of CD8^+^ T cells in AT inflammation, Nishimura et al. [14] transferred a large number of splenic CD8^+^ T cells into CD8-deficient mice and then examined the mice at 14 weeks of age. Transfer of a large number of CD8^+^ T cells increased both M1 macrophage infiltration and the expression of the proinflammatory factors IL-6 and TNF-α in epididymal fat and induced impaired glucose tolerance. The presence of CD8^+^ T cells may promote AT inflammation. However, previous studies have shown that an increase in IL-6 mRNA levels in contracting skeletal muscle is detectable after 30 min of exercise, and IL-6 mRNA levels may increase up to 100-fold at the end of an exercise period [57]. High circulating IL-6 levels can induce the production of the anti-inflammatory cytokines IL-1ra and IL-10 to promote an anti-inflammatory environment [58]. The anti-inflammatory effects of an acute exercise session protect against chronic systemic low-grade inflammation. Therefore, it is not always beneficial to inhibit the production of inflammatory cytokines to prevent AT inflammation. More studies are needed to clarify the specific pathways by which cytokines from different sources exert activity and influence corresponding diseases. To determine whether obese AT can activate CD8^+^ T cells, Nishimura cocultured CD8^+^ T cells isolated from the spleen with epididymal AT obtained from lean and obese mice and found that T cell proliferation was significantly induced by obese epididymal AT but only moderately induced by epididymal fat from lean mice, which indicates that the cellular environment of obese AT can activate CD8^+^ T cells to trigger AT inflammation and lead to a series of metabolic disorders. Furthermore, Duffaut et al. [59] showed that macrophage infiltration and AT inflammation were reduced in DIO and ob/ob mice when an anti-CD8 antibody was administered to neutralize CD8^+^ T cells or cause CD8^+^ T cell defects. This finding further shows that CD8^+^ T cells play an important role in mediating obesity-associated AT inflammation and that CD8^+^ T cells may exacerbate AT inflammation by recruiting macrophages. This amplifying effect of inflammation caused by the mutual activation of CD8^+^ T cells and AT in obese subjects further exacerbates the development and progression of inflammation.

### 3.1 CD8^+^ T cells are critical for amplifying adipose tissue inflammation

CD8^+^ T cells play an important role in the development of inflammation in obese AT. Previous studies have shown that CD8^+^ T cell activation precedes macrophage accumulation; moreover, CD8^+^ T cell activation induces macrophage activation and phenotypic changes, which trigger inflammation. Therefore, CD8^+^ T cells are an important basis of AT inflammation. In the study by Monk et al. [60] in 2015, C57BL/6 mice were fed 3% mermaid oil + 7% safflower oil (FO), which is rich in long-chain (LC) n-3 polyunsaturated fatty acids (PUFAs), or 10% caloric safflower oil as a control (CON). After 3 weeks of feeding, CD8^+^ T cells were isolated from the spleen and cocultured with 3T3-L1 adipocytes. During the coculture experiment, a physiological dose of obesity-related LPS was used to stimulate inflammation to recapitulate the inflammatory microenvironment of obese AT cells before macrophage activation. The results showed that the mRNA and secreted protein levels of TNF-α, IL-6 and MCP-1 decreased in the FO group, the activation of inflammatory transcription factors decreased, and macrophage chemotaxis in the FO group was 74% lower than that in the CON group, indicating that FO-induced changes in the paracrine interactions between CD8^+^ T cells and adipocytes affect macrophage recruitment, thereby inhibiting the development and progression of inflammation. Therefore, on the one hand, increasing FO intake may reduce inflammation in obese AT; on the other hand, FO affects the crosstalk between CD8^+^ T cells and adipocytes, inhibits the early stage of inflammation and reduces a series of metabolic complications caused by obese AT inflammation. In a 2020 study by Monk et al. [61], the role of adipokines in mediating this crosstalk and the mechanism by which adipokines affect the polarization of M1 and M2 macrophages were studied. The anti-inflammatory effect of LCn-3 PUFAs on CD8^+^ T cell/adipocyte crosstalk and the reduction in macrophage polarization may be due to the reduction in TNF-α signal transduction and the activation of downstream inflammatory mediators. Before the activation of macrophages, many factors affect the crosstalk between CD8^+^ T cells and adipocytokines in AT, and it is critical to further clarify the main factors and their mechanisms.

### 3.2 The polarized environment of obesity-associated AT supports a proinflammatory role for CD8^+^ T cells

CD8^+^ T cells mediate an inflammatory cascade in obese AT. Previous studies have shown that memory T cells exhibit an innate immune response to cytokine stimulation [62], which increases the levels of IL-12, IL-18 and IL-15 in the AT of obese mice; these Th1/T cytotoxic 1 (Tc1)-associated cytokines are produced by macrophages/dendritic cells (DCs), suggesting a Tc1/Th1-polarized environment in AT, and may be important for CD8^+^ T cell activation. Based on this hypothesis, the study by Erlie et al. [63] showed that elevated Tc1/Th1 polarized cytokine levels in the local AT environment of obese mice activated CD8^+^ T cells in AT and induced the proliferation of local CD8^+^ T cells. HFD feeding induces local inflammation in AT by releasing proinflammatory mediators such as IL-12 and IL-18, activating CD8^+^ memory T cells in AT, and inducing the polarization and proliferation of Tc1 cells, leading to the proliferation of CD8^+^ effector memory T cells/effector T cells (TEM/TE cells) in AT. This increases the infiltration of CD8^+^ T cells in AT, and these infiltrating T cells are activated by the local Tc1-polarized environment to become TEM/TE cells. Moreover, IFN-γ production is increased, which in turn activates macrophages/DCs and induces the polarization of M1 macrophages, thus forming an inflammatory cycle that amplifies inflammation in AT. These previous researchers also found that the lack of CD11a can inhibit T cell infiltration and activation in AT, resulting in the accumulation of TEM/TE cells in AT and disrupting the vicious cycle of AT inflammation, thereby reducing the activation of macrophages/DCs and reducing AT inflammation. CD11a is the alpha chain of lymphocyte functional antigen-1 (LFA-1), which can activate normal synapses and T cells by interacting with intercellular adhesion molecule-1 on endothelial cells or antigen-presenting cells. CD11a deficiency in obese mice can significantly reduce the accumulation and activation of T cells in AT. These studies demonstrate the important role of CD8^+^ T cells in initiating and amplifying obesity-associated AT inflammation and clarify the important factors that mediate this process. Targeting these factors is expected to become an important strategy to limit obesity-associated AT inflammation.

### 3.3 The proinflammatory response of CD8^+^ T cells is driven by specific antigens in adipose tissue

In addition to the above mentioned reports, a recent study showed that CD8^+^ T cell activation is associated with antigens in obese AT. As early as 2009, Feuerer et al. [40] reported that the activation and proliferation of CD8^+^ T cells in obese VAT may be driven by specific antigens that are rich in obese VAT, but the identity of these antigens was not clear. In subsequent studies, Granados et al. [64] reported that numerous peptides present on the surface of MHC class I-expressing cells, collectively known as MHC class I-associated immunopeptides (MIPs), were found to interact with CD8^+^ T cells. Furthermore, based on these findings, Chen et al. [65] used large-scale mass spectrometry to analyse VAT-derived MIPs and compared them in obese and nonobese states. The researchers reported that HFD-induced obesity induced a series of proteins, such as the MIPs lipopolysaccharide binding protein (LBP), melatonin-related receptor (GPR50) and phospholipid transfer protein (PLTP), in VA; these proteins are associated with obesity-related chronic inflammation and metabolic disorders. Therefore, obese VAT harbours a unique set of MIPs derived from obesity-related proteins that drive the proinflammatory response of CD8^+^ T cells. In addition, the researchers proved that some antigenic peptides presented by obesity-specific MHC I have immunogenicity, which may be associated with the proinflammatory response of CD8^+^ T cells. Some MHC I-restricted peptides exist only among MIPs of obese VAT and show dose-dependent immunogenicity in inducing a proinflammatory response in CD8^+^ T cells. MIPs may play roles in obesity-associated AT inflammation mediated by CD8^+^ T cells. In addition to the above mentioned antigenic peptides, there may be other antigenic peptides in VAT that trigger or maintain AT inflammation. Therefore, the analysis of MIP source proteins and the identification of key molecular targets are expected to clarify the pathogenesis of obesity and its related metabolic disorders to provide new research directions for the diagnosis and prevention of obesity and related metabolic disorders.

## 4. Effects of leptin and adiponectin on T cell function

As adipocytokines, leptin and adiponectin play important roles in the development and regulation of AT inflammation by affecting the proliferation and activation of immune cells and mediating the secretion of cytokines related to immune cells.

Leptin is an important adipokine secreted by AT and a key regulator of immunity that functions as a proinflammatory cytokine [66]. Lord et al. [67] analysed cytokines in responding cells in C57BL/6 ob/ob mice that could not produce functional leptin and found that ob/ob T cells produced a small amount of IFN-γ and an appropriate amount of IL-4 in the absence of leptin, while the addition of leptin induced considerable IFN-γ production. IL-4 production was inhibited in a dose-dependent manner. These findings suggest that leptin may bias the T cell response towards the proinflammatory phenotype. Similarly, Gerriets et al. [68] observed decreased production of IFN-γ and IL-17 and expression of key glycolytic enzymes in Th17 cells during experimental autoimmune encephalomyelitis generated by rapidly inducing hypoleptinemia, suggesting that excessive leptin can promote the secretion of immune cytokines and play a proinflammatory role. In addition, studies have shown that leptin plays a key role in controlling the proliferation of Treg cells. In vitro neutralization of leptin leads to human Treg cell proliferation, and experimental studies in leptin and leptin receptor-deficient mice showed enhanced Treg cell proliferation. In obese patients, serum leptin is proportional to AT mass, so elevated serum leptin concentrations may reduce Treg cell proliferation and promote AT inflammation [69].

Plasma adiponectin is another adipocytokine secreted by adipocytes, and its plasma concentration decreases with increasing obesity. Studies have found that adiponectin can induce IL-10 expression and production in human macrophages [70]; IL-10 is a major anti-inflammatory cytokine that can effectively inhibit the production of proinflammatory cytokines [71]. In obese AT, the plasma concentrations of adiponectin and IL-10 are significantly reduced [72], which results in a weakened anti-inflammatory effect, thus exacerbating the development of AT inflammation. In addition, adiponectin can mediate IL-10 secretion by Treg cells. Previous studies by Ramos et al. in mice showed that the lack of adiponectin receptor 1 (AdipoR1) led to the development of an obesity phenotype [73]. In recent studies, AdipoR1 was expressed on human Treg cells, and AdipoR1^+^ Tregs produced higher levels of IL-10 than AdipoR1^−^ Tregs. In addition, adiponectin may play a protective role in inflammatory conditions through the globular C-terminal fragment (gAd)/AdipoR1 axis [74]. Similarly, adiponectin can regulate CD4^+^ T cell numbers and mediate the development of inflammation. Surendar et al. [8] demonstrated for the first time that in a background of obesity, adiponectin reduces the production of IFN-γ and IL-17 by inhibiting glycolysis in Th1 and Th17 cells, thus alleviating the development of inflammation. The effects of leptin and adiponectin on immune cell function have attracted increasing attention, and further understanding of their mechanisms is expected to provide targets for the treatment of obesity.

## 5. Conclusion and prospects

Obesity occurs frequently worldwide, and patients often suffer from reduced quality of life or even life-threatening conditions due to various metabolic complications. At present, the role of immune cells in AT of obese patients has been widely examined. Previous studies have focused on macrophages, while the role of T cells in AT inflammation has gradually attracted attention in recent years. Importantly, an increasing number of studies have shown that the same cytokines secreted by T cells have both protective and proinflammatory effects on AT in obese patients. However, the mechanism is still not fully understood. Therefore, clarifying the mechanisms of T cell subtypes in AT inflammation is expected to provide new strategies for the treatment of obesity.

## Data Availability

The authors confirm that the data supporting the findings of this study are available within the article [and/or] its supplementary materials.
